# Biocontrol Agents Increase the Specific Rate of Patulin Production by *Penicillium expansum* but Decrease the Disease and Total Patulin Contamination of Apples

**DOI:** 10.3389/fmicb.2017.01240

**Published:** 2017-06-30

**Authors:** Xiangfeng Zheng, Qiya Yang, Xiaoyun Zhang, Maurice T. Apaliya, Giuseppe Ianiri, Hongyin Zhang, Raffaello Castoria

**Affiliations:** ^1^School of Food and Biological Engineering, Jiangsu UniversityZhenjiang, China; ^2^Department of Agricultural, Environmental and Food Sciences, University of MoliseCampobasso, Italy

**Keywords:** *Rhodotorula mucilaginosa*, *Rhodotorula kratochvilovae*, *Penicillium expansum*, patulin, qPCR, apples

## Abstract

Synthetic fungicides are commonly employed for the control of postharvest diseases of fruits. However, due to health concerns about the use of these chemicals, alternative control methods including biocontrol based on antagonistic yeasts are gaining in popularity. In this study, we investigated the effects of two biocontrol yeasts, *Rhodotorula mucilaginosa* strain 3617 and *Rhodotorula kratochvilovae* strain LS11, on blue mold and patulin (PAT) contamination caused by *Penicillium expansum* strains PY and FS7 in artificially inoculated Fuji apples stored at 20°C for 9 days. To correlate the development of the *P. expansum* strains in yeast-treated and untreated apples with PAT production, we quantified their biomass in the infected fruits using a recently published quantitative real-time polymerase chain reaction method based on specific primers for patF, a gene from *P. expansum* that is involved in PAT biosynthesis. Both yeasts significantly reduced the disease incidence caused by the two strains of *P. expansum* up to 5–7 days of incubation, and lowered their biomass and the progression of symptoms up to 9 days. Interestingly, both yeasts strains increased the rate of PAT production (expressed as ng patulin/μg fungal DNA) by the two pathogenic strains. Nevertheless, both biocontrol agents reduced the total PAT contamination, especially in the case of *P. expansum* strain FS7, the higher PAT producer of the two tested *P. expansum* strains. Comparing between the yeast strains, *R. kratochvilovae* LS11 was more effective than *R. mucilaginosa* 3617 for the control of *P. expansum*.

## Introduction

The contamination of food and feed with mycotoxins, which are toxic secondary metabolites produced by plant pathogenic fungi, poses a serious health hazard to consumers (Capcarova et al., 2016). The mycotoxin patulin (PAT) is produced by some *Penicillium*, *Aspergillus*, and *Byssochlamys* species. In mammals, the primary target organs of PAT toxicity are the kidney, liver, immune system and gastrointestinal tract. There is a lack of evidence for PAT carcinogenicity in humans and experimental animals, and this mycotoxin is placed in group 3 by the International Agency for Research on Cancer (IARC, 1986). However, the long-term consequences of exposure to toxic PAT concentrations include mutagenicity, genotoxicity, and embryotoxicity, while the effects of exposure to high dosages include immunosuppression, immunotoxicity, and neurotoxicity (Glaser and Stopper, 2012).

The fungal species *Penicillium expansum* is a major PAT-producing pathogen of stored fruits, especially pome fruits and derived products (Saladino et al., 2016). PAT contamination poses a major risk for children, who consume large quantities of fruit juices. Therefore, regulations have been released worldwide, that establish specific limits for PAT contamination in fruit-derived products, as in the case of the European Union which legislates that the highest tolerable levels of this mycotoxin are 50 μg/kg in fruit- derived products and 10 μg/kg in baby food (Commission EC, 2006).

Several studies have reported on the degradation and/or detoxification of PAT or the reduction of its production in fruits (Assatarakul et al., 2012; Funes et al., 2013; Yue et al., 2013). The utilization of antagonistic microorganisms as biocontrol agents (BCAs) is promising because such agents have no known toxicity toward human health or ecosystem (Castoria et al., 2011; Zhu et al., 2015a). We previously showed that two antagonist basidiomycetous yeasts, *Rhodotorula mucilaginosa* strain 3617 and *Rhodotorula kratochvilovae* strain LS11 [previously reported as *Rhodosporidium kratochvilovae*, and recently reclassified (Wang et al., 2015)], are effective BCAs against the blue mold and PAT contamination of stored apples caused by *P. expansum* (Castoria et al., 2005; Yang et al., 2015). These two BCAs can degrade PAT *in vitro*, as also reported for other basidiomycetous yeasts such as *Rhodosporidium paludigenum* and *Sporobolomyces* sp. (Castoria et al., 2011; Yang et al., 2015; Ianiri et al., 2017). The transcriptomic profile of *Sporobolomyces* sp. in the presence of PAT has also been reported (Ianiri et al., 2016). However, it must be emphasized that PAT degradation by BCAs has not yet been shown *in vivo*, i.e., in apples infected by *P. expansum*.

Recently, Zhu et al. (2015b) showed that in the low percentage of infected apples and pears pretreated with the yeast *R. paludigenum* as a BCA, PAT accumulation was increased compared with infected control fruits without *R. paludigenum* pretreatment. These authors hypothesized that the stress caused to *P. expansum* by the presence of *R. paludigenum* the BCA and the low PAT-degrading capability of this yeast in the rotting apple tissue could explain the increased PAT accumulation. Conversely, our studies and other studies have observed decreases of mycotoxin accumulation following treatment with BCAs as compared to untreated controls (Magan et al., 2011; Cao et al., 2013; Spadaro et al., 2013). There is a need to shed light on these conflicting results by assessing the possible stress effect of BCAs on *P. expansum* and its possible consequences on PAT biosynthesis *in vivo*, i.e., in stored pome fruits. This can be achieved by extending the investigations to a greater number of pathogen strains, BCAs, and apple cultivars, and by determining the rate of PAT production per unit of fungal biomass. So far, studies on BCAs have evaluated *P. expansum* growth in infected fruits based on the development of disease symptoms such as lesion diameter or rotting volume (Baert et al., 2007; Li et al., 2011). Thus, there is little quantitative information about the effects of postharvest BCAs on fungal growth and PAT production by *P. expansum* in apples.

In this study, we compared the efficacies of the biocontrol yeasts *R. mucilaginosa* strain 3617 and *R. kratochvilovae* strain LS11 which were isolated in China and Italy in controlling blue mold decay on stored Fuji apples artificially inoculated with *P. expansum* strains PY and FS7, which were isolated from the same geographic locations, respectively. Furthermore, we used a recently developed quantitative real-time polymerase chain reaction (qPCR) approach based on specific primers to patF, a gene from *P. expansum* that is involved in PAT biosynthesis (Tannous et al., 2015), to quantitatively determine the influence of the BCAs on the dynamics of *P. expansum* biomass. This also allowed us to assess the specific rate of PAT production by the *P. expansum* strains in infected apples either with or without pretreatment using the biocontrol yeast strains.

## Materials and Methods

### Biocontrol Agents

The biocontrol yeast *R. mucilaginosa* strain 3617 (preserved in the China General Microbiological Culture Collection Center, Accession number 3617) was isolated from the surface of peach blossoms in a chemically untreated orchard in China. The biocontrol yeast *R. kratochvilovae* LS11 was obtained from the culture collection of the Department of Agricultural, Environmental and Food Sciences, at the University of Molise, Italy. Both BCAs were routinely maintained at 4°C on nutrient yeast dextrose agar [NYDA: 8 g nutrient broth, 5 g yeast extract, 10 g glucose, and 20 g agar (Sangon Biotech, Shanghai, China)] in 1 L distilled water. Liquid cultures of the yeasts were prepared in 250-mL Erlenmeyer flasks containing 50 mL of nutrient yeast dextrose broth (NYDB) that had been inoculated with cells withdrawn with a loop from the agar medium mentioned above. Cellular suspensions of *R. mucilaginosa* 3617 or *R. kratochvilovae* LS11 were incubated for 20 h on a rotary shaker at 180 rpm, at 28 and 23°C, respectively. Following incubation, the cultures were centrifuged at 7000 ×*g* (TGL-16M Centrifuge, Xiangyi Co., Changsha, China) for 10 min and washed twice with sterile distilled water. Yeast cell pellets were suspended in sterile distilled water and their concentrations were adjusted with a hemocytometer to 1 × 10^8^ cells mL^-1,^ as required for the subsequent experiments.

### Pathogens

*Penicillium expansum* strain PY (preserved in the China General Microbiological Culture Collection Center, accession number 3703) was isolated from infected apples in China, while *P. expansum* strain FS7 was obtained from the Culture Collection Center of the Department of Agricultural, Environmental and Food Sciences, at the University of Molise, Italy. The fungal cultures were maintained on potato dextrose agar (PDA: 200 g extract of boiled potatoes, 20 g glucose, 20 g agar, and 1 L distilled water) at 4°C. Fresh cultures were grown on PDA plates at 25°C before use. Spore concentrations were determined with a hemocytometer, using sterile distilled water for the adjustments.

### Apple Fruits

Apples (*Malus domestica* Borkh, cv. Fuji) were harvested at commercial maturity from an orchard in Yantai, Shandong province, China, and selected based on their uniformity of size, ripeness, and absence of apparent injury or infection. Fruits were surface disinfected with 0.1% (w/v) sodium hypochlorite for 1 min, washed with tap water and allowed to dry at room temperature.

### Assessment of Biocontrol Activity of *R. mucilaginosa* 3617 and *R. kratochvilovae* LS11

Apples were wounded with a sterile cork borer (approximately 3-mm-diameter and 3-mm-deep). In each wound, 30 μL of the following suspensions were alternatively applied: (1) *R. mucilaginosa* 3617 (1 × 10^8^ cells mL^-1^), (2) *R. kratochvilovae* LS11 (1 × 10^8^ cells mL^-1^), (3) sterile distilled water as a control. Two hours later, 30 μL of *P. expansum* (PY or FS7) suspensions (5 × 10^4^ spores mL^-1^) were inoculated into each wound. After air drying, the samples were stored in enclosed plastic trays to maintain a high relative humidity (about 95%) and incubated at 20°C in an incubator (Radford Technology Co., Ltd., Ningbo, China). The disease incidence and symptoms, expressed as the percentage of infected fruit wounds and diameter of lesions in mm, respectively, were recorded at 3, 5, 7, and 9 days after inoculation. The experiment was conducted twice. Each experiment consisted of three replicates per time point, and each replicate consisted of nine apples, each with three wounds. Data from the two experiments were similar as resulting from ANOVA analysis and were pooled. Bars in the figure represent mean values ± standard deviations.

### Effects of the Biocontrol Agents *R. mucilaginosa* 3617 and *R. kratochvilovae* LS11 on the Contamination of Apples with PAT

Patulin production by strains PY and FS7 of *P. expansum* in rotting apple tissues was assessed as previously described (Castoria et al., 2011; Yang et al., 2015) with slight modifications. Samples for the analysis of PAT were collected and extracted on days 5, 7, and 9 after inoculation. In order to consistently represent the expected progression of the fungal disease over time (i.e., in terms of increase of lesion size and of fungal biomass), the samples (infected fruit wounds) that were chosen for withdrawal on days 7 and 9 were the ones that at the respective previous time points (day 5 for day 7, and day 7 for day 9) had similar lesion diameters to those of samples that were withdrawn on days 5 and 7, respectively. After withdrawing the rotten tissue and 1 cm of surrounding healthy tissue using a sterile cork borer from infected wounds, the latter were pooled and homogenized by using a homogenizer (TTL-260, Beijing TongTaiLian Technology co., Ltd., Beijing, China) with rotor speed set at 15000 rpm. Afterward, each sample was weighed and was split into samples of 20g each, one used for PAT extraction and analysis, and the other 20 g was used for DNA quantification (see below). The sample for PAT determination was mixed with 25 mL of sterilized distilled water and transferred into a 50 mL conical flask. The samples were then treated overnight at room temperature with 100 μg mL^-1^ of pectinase (500 units/mg, Sangon Biotech). Afterward, the same volume of ethyl acetate was added to each sample and shaken vigorously for 5 min. The upper layer was then transferred into a separatory funnel. This process was repeated twice and the organic phases of each sample were pooled. Ten milliliters of 14 g L^-1^ sodium carbonate solution were then added to the organic phases and shaken for 10 s. The phases were allowed to settle, then they were separated and the aqueous phase was immediately extracted with 10 mL of ethyl acetate by shaking for 1 min. Subsequently, five drops of glacial acetic acid were added to the organic phases, which were then evaporated to dryness in a water bath at 40°C. The resulting residue was immediately dissolved in 1 mL of acetonitrile/water (1:9 v/v), the mobile phase used for HPLC analyses, and filtered through a 0.22 μm filter (WondaDisc NY organic filter, SHIMADZU-GL Sciences, Shanghai, China). Finally, 20 μL of each filtered solution was injected into a high-performance liquid chromatography (HPLC) apparatus to determine the PAT content. Agilent 1260 series system equipped with a quaternary pump and variable wavelength detector (Agilent Technologies, Santa Clara, CA, United States) was used with a Zorbax^®^ analytical column (SB-C_18_ 250 mm × 4.6 mm, 5 μm, Agilent Technologies). The mobile phase was acetonitrile/water (1:9 v/v) with a flow rate of 1 mL min^-1^. Detection was performed by measuring the absorbance of UV light at 276 nm.

Each experiment consisted of three replicates per time point, and each replicate consisted of three apples, each with three wounds. Rotting wounds collected from each replicate were pooled before extraction. The experiment was conducted twice. Data from the two experiments, which were expressed as μg patulin/g of decayed apple tissue, were similar as resulting from ANOVA analysis and were pooled. Bars in the figure represent mean values ± standard deviations.

### Extraction of Genomic DNA from Biocontrol Yeasts, Fungal Pathogens, and Healthy Apple Fruits

The yeasts were cultivated on NYDA and a single colony was picked out and transferred to NYDB. After 48 h of growth at 28 and 23°C (for *R. mucilaginosa* 3617 and *R. kratochvilovae* LS11, respectively) with agitation at 180 rpm, the yeast cells were harvested for DNA extraction. Strains FS7 and PY of *P. expansum* were freshly grown on PDA for 7 days at 25°C. Afterward, the spores were withdrawn, their concentration was adjusted to 10^5^ spores mL^-1^, and they were added to a 250-mL Erlenmeyer flask containing 100 mL of PDB that was maintained under constant agitation at 180 rpm for 4 days at 25°C. Genomic DNA of the yeast strains, *P. expansum* strains, and healthy apple fruits were extracted using the method described by Ihrmark et al. (2012). The yeast cells, the fungal mycelia and the apples were collected and freeze-dried. Two hundred milligrams of fungal mycelia or yeast cells were ground into powder with liquid nitrogen. The resulting powder was collected into micro-centrifuge tubes to which 700 μL of lysis buffer CTAB [2% hexadecyltrimethylammonium bromide (w/v), 1.4 M NaCl, 20 mM ethylenediaminetetraacetic acid pH 8, 100 mM Tris-HCl pH 8] was added. The lysis mixtures were incubated at 65°C for 1 h and then cooled on ice for 1 h. Afterward, the samples were treated with 500 μL of phenol: chloroform (1:1 v/v) and vortexed for 1 min and the supernatant was centrifuged at 13,000 rpm for 15 min at 4°C. Genomic DNA was precipitated with an equal volume of ice-cold isopropanol by incubation overnight at -20°C. After incubation, the samples were centrifuged at 13,000 rpm for 10 min at 4°C. The pellets were thoroughly rinsed with 70% ethanol, air-dried and re-suspended in 50 μL of sterile distilled water. The DNA purity ratios and concentrations were measured using a NanoDrop 2000 Spectrophotometer (Thermo Fisher Scientific, Waltham, MA, United States). Extraction of genomic DNA from yeasts, fungi and healthy apples was performed to assess the specificity of the primers chosen for the quantification of *P. expansum* biomass through qPCR.

### Assessment of Specificity of Primers for qPCR

For the assessment of the development of the pathogenic and mycotoxigenic *P. expansum* strains PY and FS7 in apple fruits, we used a primer pair specific for *P. expansum PatF* (GenBank Accession No. AIG62137), a gene involved in PAT biosynthesis: patF_F (ATGAAATCCTCCCTGTGGGTTAGT) and patF_R (GAAGGATAATTTCCGGGGTAGTCATT), designed by Tannous et al. (2015). The primers were synthesized by Sangon Biotech and their specificity was tested by carrying out polymerase chain reaction (PCR) experiments with genomic DNA from the *P. expansum* strains, the BCAs, and healthy Fuji apples as templates. All PCR amplifications were performed in a total reaction volume of 25 μL consisting of 2 μL DNA template (∼50 μg), 2.5 μL 10 × PCR buffer (containing Mg^2+^), 2.5 μL dNTPs (2.5 μM each), 2.5 μL of each primer (0.5 μM each), 0.2 μL Taq DNA polymerase (LA taq TAKARA, Japan), and H_2_O up to 25 μL. The PCR conditions involved five steps: 94°C for 5 min, 94°C for 30 s, 60°C for 30 s, 72°C for 10 s, 72°C for 10 min. A total of 40 cycles of amplification were performed from steps 2–4. The PCR results were analyzed by agarose gel (1% w/v) electrophoresis.

### DNA Extraction from Yeast-Treated and Untreated Infected Apples for Assessing the Development of *P. expansum* Strains FS7 and PY

DNA extractions from apples infected by *P. expansum* strains, both treated and untreated with the BCAs were performed at days 5, 7, and 9 after the inoculation of the fruits. The extractions of genomic DNA for qPCR analysis were performed from 20 g portions of the same samples as those used for PAT extraction and analysis. Extractions were performed according to the protocol described by Rodriguez et al. (2011) with slight modifications. Fifty milliliters of Tris-HCl (pH 8) were added to 20 g of homogenized sample and then vortexed for homogenization. The sample was filtered through gauze paper and then centrifuged at 10,000 rpm for 5 min. The resulting pellet was suspended in 100 μL of pre-heated sterile distilled water and then cooled on ice for 10 min. Five hundred microliters of lysis buffer (2% hexadecyltrimethylammonium bromide, 1.4 M NaCl, 20 mM ethylenediaminetetraacetic acid EDTA pH 8, 100 mM Tris-HCl pH 8) and 20 μL of proteinase K (Sangon Biotech) were added. The sample was incubated at 65°C for 1 h and then centrifuged at 10,000 rpm for 5 min. The supernatant was transferred to a new centrifuge tube containing 500 μL phenol/chloroform (1:1 v/v). The solution was thoroughly mixed and then centrifuged at 13,000 rpm for 10 min at 4°C. The upper layer was carefully transferred to a new centrifuge tube and 10 μL of RNase solution (Sangon Biotech) were added before incubation at 37°C for 1 h. Afterward, an equal volume of chloroform was added, the mix was vortexed and centrifuged at 13,000 rpm for 5 min. The top layer was transferred to a new microcentrifuge tube, DNA was precipitated with an equal volume of ice-cold isopropanol and incubated at 20°C. The obtained pellet was rinsed with 70% ethanol, air-dried and suspended in 30 μL sterile distilled water. The DNA purity ratio and concentration were measured as described above. Since the experiments for the quantification of fungal DNA were performed with aliquots of the same samples as those collected for PAT quantification, they consisted of the same number of replicates, apples and wounds. Fungal biomass was expressed as ng DNA/μg decayed apple tissue. All the genomic DNA sample used in this work were also tested for their suitability for PCR amplification using primers actin-F and actin-R (actin-F: TCCTTCGTCTTGACCTTGCT, actin-R: ACTTCATGATGGAGTTGTAGGTAGT) (Sangon Biotech) on the basis of the actin gene sequence of *Malus pumila* (Hightower and Meagher, 1986).

### Quantification of *P. expansum* Biomass through qPCR

qPCR assays with DNA samples extracted from infected apples were performed to determine fungal biomass using SYBR Premix Ex Taq TM II (Tli RNaseH Plus) (TAKARA, Japan) with the Bio-Rad CFX96 (Bio-Rad, Hercules, CA, United States) and the primer pair patF_F and/patF_R. Two negative controls were also performed (one without primers and fungal DNA and the other one without fungal DNA) to rule out any possible matrix effect. The PCR conditions were as follows: 94°C for 30 s, 40 cycles of 94°C for 20 s, 60°C for 20 s, and 72°C for 20 s. The dissociation curve analysis followed the same trend as the amplification cycle and was constructed by continuously measuring the fluorescence when increasing the temperature from 65 to 95°C, at the rate of 0.5°C/s. The threshold cycle (CT) values were automatically determined by the CFX Manager^TM^ software (Bio-Rad Laboratories). For each DNA sample, three technical replicates were analyzed. Fungal biomass was expressed as ng DNA/μg decayed apple tissue.

### Calculation of Specific Mycotoxigenic Activity

The specific rates of PAT biosynthesis (specific mycotoxigenic activity) by *P. expansum* strains PY and FS7 were calculated using the mean concentration of PAT and *P. expansum* DNA in decayed apple tissue as measured in the two experiments and expressed as ng patulin/μg fungal DNA.

### Statistical Analysis

The percentages of apple wounds infected with *P. expansum* were converted to Bliss angular values (arcsine square root) before analysis of variance. Data were subjected to analysis of variance (ANOVA, SPSS release 17.0 for Windows; SPSS Inc., Chicago, IL, United States). All the means were compared by using Duncan’s multiple range test (*P* < 0.05). All the experiments were performed twice.

## Results

### Biocontrol of Blue Mold Decay by *R. mucilaginosa* 3617 and *R. kratochvilovae* LS11

**FIGURE 1:**
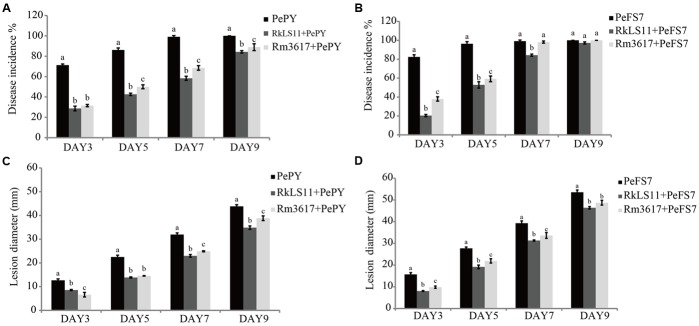
Time course of biocontrol activity of *Rhodotorula mucilaginosa* 3617 and *Rhodotorula kratochvilovae* LS11 against blue mold decay of apples. **(A)** Decay incidence (%) of infected wounds caused by *Penicillium expansum* strain PePY. **(B)** Decay incidence (%) caused by *P. expansum* strain PeFS7. **(C)** Lesion diameter (mm) of apples infected by *P. expansum* strain PePY. **(D)** Lesion diameter of apples infected by *P. expansum* FS7. PePY, *P. expansum* strain PY; PeFS7, *P. expansum* strain PeFS7; Rm3617, *Rhodotorula mucilaginosa* strain 3617; RkLS11, *R. kratochvilovae* strain LS11. Bars represent the mean values from two experiments ± standard deviations. Values marked with different letters are significantly different (*P* < 0.05).

The two biocontrol yeasts significantly reduced the percentage of infected wounds in apples inoculated with *P. expansum* strain PY (**Figure 1A**). At days 3, 5, 7, and 9 after inoculation, yeast strain LS11 reduced the disease incidence caused by *P. expansum* strain PY by 59.7, 50.5, 41.1, and 15.7%, respectively. In apples treated with yeast strain 3617 the disease incidence was reduced by 55.8, 41.9, 30.9, and 11.1% at the same time intervals (**Figure 1A**). When the BCAs were challenged by *P. expansum* strain FS7, no decrease of disease incidence was recorded after day 5 in the apples pretreated with *R. mucilaginosa* 3617 or after day 7 in apples pretreated with *R. kratochvilovae* LS11. Strain 3617 reduced the disease incidence by 53.9 and 38.4% at days 3 and 5, respectively, while strain LS11 reduced the disease incidence 75.2, 45.2, and 14.9% after 3, 5, and 7 days, respectively (**Figure 1B**).

Furthermore, both BCAs caused significant reductions in the mean diameters of the lesions caused by both strains of *P. expansum* at all tested time intervals, although this effect was less pronounced at days 5 and 7, especially in the apples pretreated with *R. mucilaginosa* 3617 (**Figures 1C,D**). In apples infected by *P. expansum* strain PY, yeast strain 3617 reduced the lesion diameters by 47.7, 35.4, 22.0, and 11.4% on days 3, 5, 7, and 9, respectively. On the same days, yeast LS11 reduced the lesion diameters by 32.3, 38.5, 38.1, and 20.5%, respectively, as compared to untreated control apples (**Figure 1C**). When the BCAs were challenged by *P. expansum* strain FS7, yeast strain 3617 decreased the lesion diameters by 37.4, 21.0, 14.3, and 9.0% on days 3, 5, 7, and 9, respectively. Yeast strain LS11 reduced lesion diameters by 48.8, 30.9, 20.3, and 13.4% at the same time intervals, respectively, as compared to the untreated control apples (**Figure 1D**).

### Effects of *R. mucilaginosa* 3617 and *R. kratochvilovae* LS11 on Patulin Accumulation in Apples Infected by *P. expansum*

**FIGURE 2:**
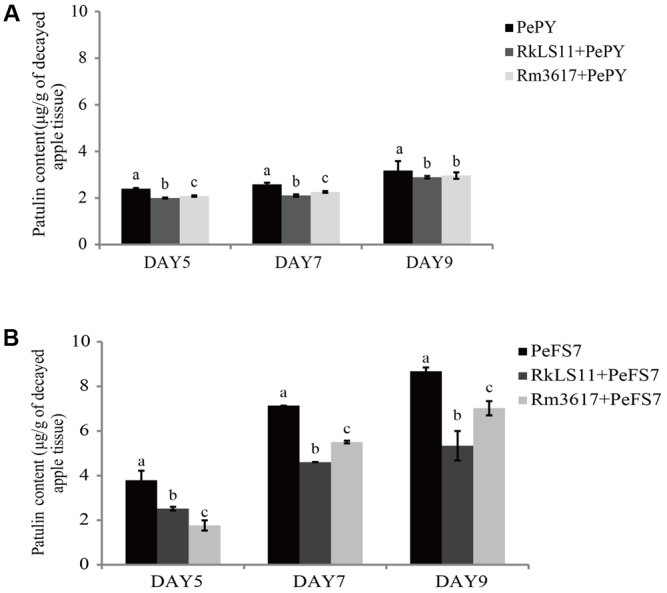
Time course of patulin (PAT) (μg/g) accumulation in apples infected by strains PePY and PeFS7 in the presence and in the absence of the biocontrol yeasts *Rhodotorula mucilaginosa* strain 3617 and *R. kratochvilovae* strain LS11. **(A)** PAT accumulation caused by *Penicillium expansum* strain PePY. **(B)** PAT accumulation caused by *P. expansum* strain PeFS7. Bars represent the mean values from two experiments ± standard deviations. Values marked with different letters are significantly different (*P* < 0.05).

The concentration of PAT that accumulated in rotted apple tissue infected with *P. expansum* PY and FS7 are shown in **Figures 2A,B**, respectively.

The PAT accumulation due to *P. expansum* FS7 was more than twice as high as that due to *P. expansum* PY at almost all of the tested time intervals. In apples infected with *P. expansum* FS7, a progressive increase of PAT contamination was observed throughout the experiment, ranging from 3.8 μg patulin/g infected apple tissue on day 5 to 8.7 μg/g on day 9 (**Figure 2B**). Conversely, the lower PAT accumulation that was recorded in apples infected with *P. expansum* PY was similar on days 5 (2.4 μg/g) and 7 (2.6 μg/g), and reached its highest value (3.2 μg/g) on day 9 (**Figure 2A**). Significant reductions of PAT contamination were evident at all tested time points in the infected apples pre-treated with both yeast strains (**Figures 2A,B**). The BCA LS11 decreased PAT accumulation due to *P. expansum* PY by 16.7, 19.2, and 9.4% on days 5, 7, and 9, respectively. Those decreases in PAT accumulation were significantly higher than those recorded in apples pretreated with strain 3617 on days 5 and 7, whereas on day 9 there was no significant difference between the apples pre-treated with the two BCAs (**Figure 2A**). The percentage decreases of PAT accumulation yielded by the two BCAs were more pronounced in apples inoculated with *P. expansum* FS7, the higher PAT producer, at all tested time points. In addition, the BCA strain LS11 yielded significantly greater decreases in PAT accumulation than strain 3617 at almost all tested time points. Strain LS11 yielded decreases in PAT accumulation of 34.2, 35.2, and 39.1% at days 5, 7, and 9, respectively, versus decreases of 52.6, 22.5, and 19.5% obtained by strain 3617 at the same time intervals, as compared to untreated control apples (**Figure 2B**).

### Specificity of Primers for the Measurement of *P. expansum* Biomass

**FIGURE 3:**
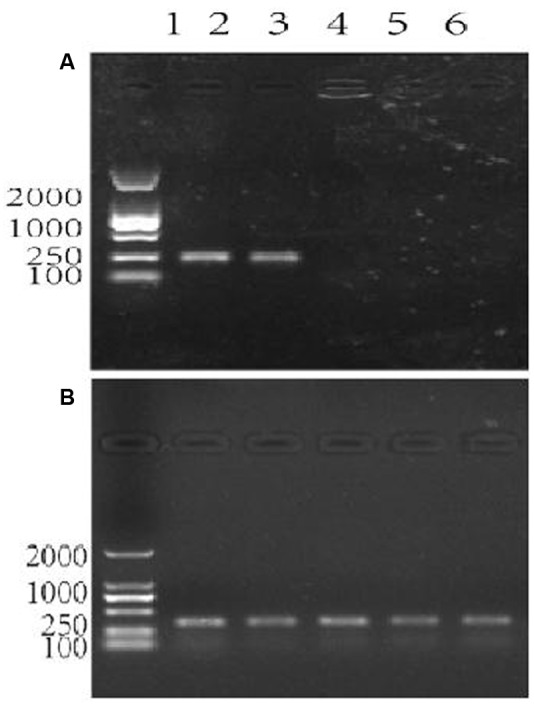
Agarose gel electrophoresis showing the assessment of specificity of the primer pair patF-F/patF-R used for the quantification of fungi (*Penicillium expansum*) DNA. Lane 1 in **(A,B)**: DNA 100-2000 Marker, Lanes 2–6 in **(A,B)**: DNA templates. **(A)** The primer pair used in the PCR reactions was patF-F/patF-R. DNA from PCR reactions in which different genomic DNAs were used as the templates were: lane 2: genomic DNA from *P. expansum* strain FS7; lane 3: genomic DNA from *P. expansum* strain PY; lane 4: genomic DNA from *R. kratochvilovae* strain LS11; lane 5: genomic DNA from *R. mucilaginosa* 3617, lane 6: genomic DNA from apple fruits cv. Fuji. **(B)** The primer pair used in the PCR reactions was ACTIN-F/ACTIN-R. Lanes 2–6 were loaded with DNA samples from PCR reactions in which the same genomic DNA templates as in **(A)** were used.

The data shown in **Figure 3A** confirmed that the primers patF-F and patF-R are specific to the genomic DNA of *P. expansum*. The primers annealed to the genomic DNA of strains PY and FS7, yielding the expected PCR product of 250 bp in each case. Conversely, no PCR products were obtained when genomic DNA samples from the two biocontrol yeast strains (lanes 5–6) and the Fuji apple fruits used in this study (lane 6) were used as templates (**Figure 3A**). **Figure 3B** shows the PCR positive control, in which the primers actin-F and actin-R were used with genomic DNA samples from the two *P. expansum* strains, the two biocontrol yeasts, and Fuji apple fruits. The DNA bands were of the expected size (330 bp), thus confirming that the quality of the genomic DNA samples used in this study was sufficient for PCR.

### Suitability of qPCR Assay for the Quantification of *P. expansum* PY and FS7 DNA

**FIGURE 4:**
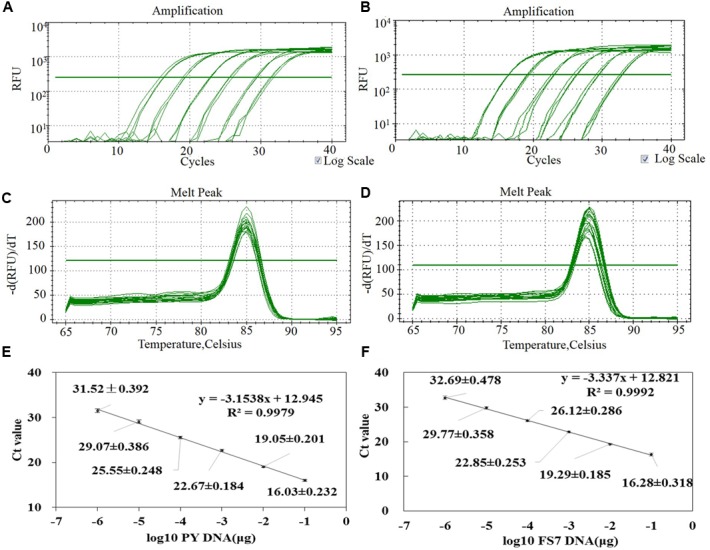
Assessment of the method for the quantification of *P. expansum* genomic DNA through qPCR based on the primers pair patF-F/patF-R. **(A,B)** Amplification curves of a set of six 10-fold serial dilutions of genomic DNA from strains PY and FS7 of *P. expansum* showing the fluorescence signal plotted versus log of PCR cycle number (blank controls were also performed but fluorescence signal was observed). **(C,D)** Dissociation curve of the PCR product. **(E,F)** Standard curve generated by qPCR assay using 10-fold serial dilutions of pure genomic DNA from strains PY and FS7 of *P. expansum*; Ct values were obtained for each dilution and plotted versus known quantities of genomic DNA used in the analyses.

After confirming the specificity of the primers patF-F and patF-R (see **Figures 3A,B**), the analytical sensitivity of the qPCR assay was determined using serial 10-fold dilutions, ranging from 1 μg to 1 pg of pure genomic DNA from *P. expansum* strains PY and FS7 of. For each sample dilution, the fluorescence emission was measured and plotted against the log of Ct values (**Figures 4A,B**). The detection limit of the assay for both *P. expansum* strains was 1 pg of DNA. The dissociation curve analyses performed after the last amplification step detected a single PCR product with a specific melting temperature of 85°C (**Figures 4C,D**), since no primer dimers were generated during the qPCR amplification. The *R*^2^ values of the linear correlations were 0.9979 and 0.9992 for strains PY and FS7, respectively (**Figures 4E,F**), and the slopes of the linear regression curves were -3.1538 and -3.337, respectively.

### Quantification of *P. expansum* Biomass in Infected Apples through qPCR

**FIGURE 5:**
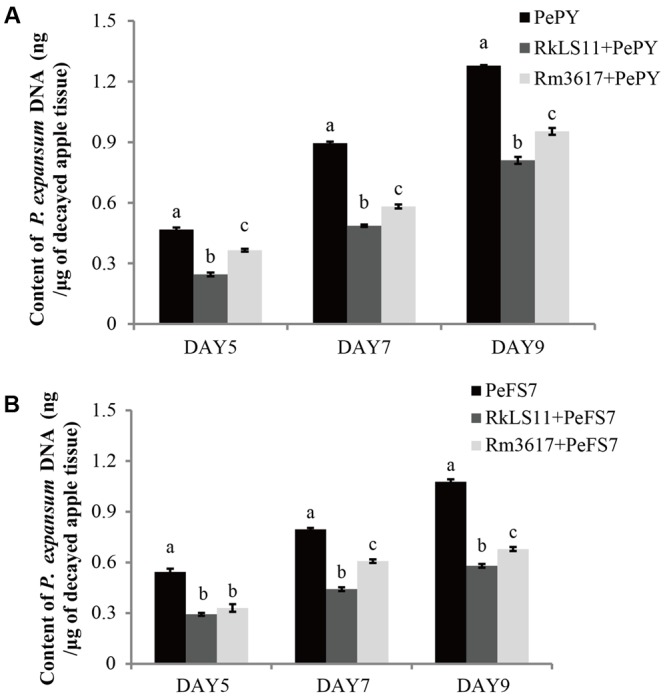
Time course quantification of DNA of *P. expansum* strains PY and FS7 in infected apples pretreated and untreated with the BCAs *R. mucilaginosa* 3617 and *R. kratochvilovae* LS11. Concentrations of DNA were expressed as ng of fungal DNA/mg of decayed apple tissue. **(A)** DNA of *P. expansum* strain PY. **(B)** DNA of *P. expansum* strain FS7. PePY, *P. expansum* strain PY; PeFS7, *P. expansum* strain PeFS7; Rm3617, *Rhodotorula mucilaginosa* strain 3617; RkLS11, *R. kratochvilovae* strain LS11. Bars represent the mean values from two experiments ± standard deviations. Values marked with different letters are significantly different (*P* < 0.05).

In infected apples that were not pre-treated with the BCAs, the biomass of both *P. expansum* strains, as measured in terms of DNA content appeared to steadily increase from day 5 to day 9 following the inoculation of fruits. The calculated biomass of *P. expansum* ranged from 0.47 to 1.28 μg/mg of infected apple tissue for PY and from 0.54 to 1.08 μg/mg for FS7 (**Figures 5A,B**).

In infected apples pre-treated with the BCAs LS11 and 3617, the biomass of both *P. expansum* strains increased at the same time intervals as in the non-pretreated apples, but at significantly lower rates (**Figures 5A,B**). Furthermore, the yeast strains LS11 and 3617 appeared to cause similar decreases in the DNA contents of *P. expansum* strains PY and FS7, although LS11 yielded greater reductions at almost all tested time points. The reductions of *P. expansum* strain PY DNA content in infected of apple tissue achieved by LS11 were 46.8, 46.7, and 36.2% at 5, 7, and 9 days after inoculation, respectively. In comparison, yeast strain 3617 yielded reductions in DNA content of 23.4, 35.6, and 25.8% at the same time intervals, respectively (**Figure 5A**). In fruits challenged with *P. expansum* FS7, the DNA content of this strain was reduced by 46.3, 45.0, and 47.3%, by LS11 on days 5, 7, and 9, respectively, whereas3617 yielded reductions in DNA content of 38.9, 23.8, and 37.0% on the same days (**Figure 5B**).

### Specific Mycotoxigenic Activity of *P. expansum* Strains PY and FS7 in Infected Apples Pretreated with BCAs

**FIGURE 6:**
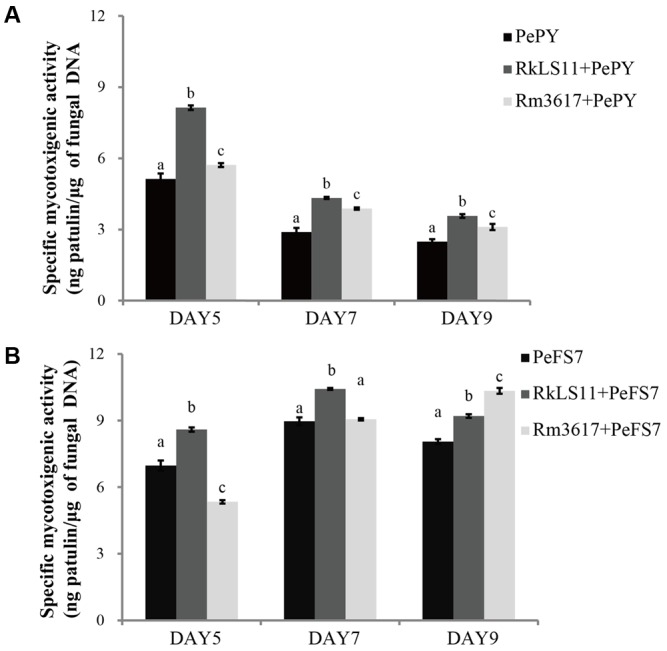
Time course of specific mycotoxigenic activity (ng patulin/μg of fungal DNA) of strains PY **(A)** and FS7 **(B)** of *Penicillium expansum* in infected apples, in the presence and in the absence of the biocontrol yeasts *Rhodotorula mucilaginosa* strain 3617 and *R. kratochvilovae* strain LS11. **(A)** Decay incidence caused by *Penicillium expansum* strain PY. **(B)** Decay incidence caused by *P. expansum* strain FS7 on apples. PePY, *P. expansum* strain PY; PeFS7, *P. expansum* strain PeFS7; Rm3617, *Rhodotorula mucilaginosa* strain 3617; RkLS11, *R. kratochvilovae* strain LS11. Bars represent the mean values from two experiments ± standard deviations. Values marked with different letters are significantly different (*P* < 0.05).

**Figures 6A,B** show the results for the pretreatment of apples with the BCAs on the specific rates of PAT biosynthesis (specific mycotoxigenic activity) by *P. expansum* strains PY and FS7 in the infected fruits. The rationale for these measurements was to assess whether the BCA enhance PAT biosynthesis owing to the competition and stress they exert on *P. expansum* in the infected wounds. The specific mycotoxigenic activity by *P. expansum* PY decreased from day 5 (5.1 ng PAT/μg DNA) to days 7 and 9 (2.9 and 2.5 ng PAT/μg DNA, respectively) (**Figure 6A**). In comparison, an approximately constant rate of PAT biosynthesis was observed for strain FS7 (7.0, 8.0, and 8.1 ng PAT/μg DNA at days 5, 7, and 9, respectively) (**Figure 6B**). Both strains 3617 and LS11 significantly increased PAT biosynthesis by *P. expansum* at all tested time points (**Figures 6A,B**), except for *P. expansum* strain FS7 in the presence of 3617 at days 5 and 7, when the biosynthetic rates were lower than and similar to the untreated controls, respectively. Increases in the biosynthetic rate of 58.8, 51.7, and 44.0% were observed at days 5, 7, and 9, respectively, for *P. expansum* PY in the presence of the yeast strain LS11, and 11.8, 34.5, and 24% at the same time points in the presence of the yeast strain 3617. In apples infected with *P. expansum* FS7, increases in the biosynthetic rate of 22.9, 15.6, and 13.6% were detected at days 5, 7, and 9, respectively, in the presence of the yeast strain LS11, and of 27.2% at day 9 for *P. expansum* FS7 in the presence of strain 3617.

## Discussion

Patulin has frequently been detected in apple juice because of the utilization of contaminated fruits, which are the result of the decay caused by *P. expansum* mainly during storage (Spadaro et al., 2007; Marin et al., 2013). This mycotoxin represents a serious health hazard, especially because fruit juices are frequently consumed by children. Consequently, regulatory bodies worldwide have set maximum tolerable limits for PAT in fruit-derived products (Commission EC, 2006; Ministry of Health of the People’s Republic of China, 2011).

At present, the control of postharvest diseases is mainly based on the utilization of synthetic fungicides. However, health and environmental concerns raised by consumers, as well as the onset of fungicide-resistant pathogen strains call for alternative control methods. Biocontrol is a promising technology for lowering chemical inputs in the food chain during cultivation and storage. However, during storage, where environmental factors are much more under human control than in the field, biocontrol cannot be considered as a “silver bullet” or panacea but rather as a part of an integrated strategy comprising multiple approaches (Wisniewski et al., 2016). Nevertheless, it is worthwhile to study the mechanisms of action of BCAs so that their activity can be potentiated, and to foster the development of commercial formulations and methods for their practical utilization (Massart et al., 2015; Di Francesco et al., 2016; Droby et al., 2016; Spadaro and Droby, 2016). Among these mechanisms, the influence of BCAs on mycotoxin contamination is a key to ensuring the proper implementation of biocontrol. Since the paper published in 2005 by Castoria et al. (2005), increasing attention has been paid to the utilization of postharvest BCAs against the mycotoxin contamination of fruits, especially pome fruits. Most of these studies point to a general reduction of PAT accumulation in stored apples that were treated with different BCAs. On the other hand, mycotoxins are secondary metabolites that are synthesized by fungi under stressful conditions. PAT has been suggested to play a role in inter-microbial competition (Reverberi et al., 2010). Therefore, the stimulation of its synthesis by *P. expansum* in the presence of competing BCAs could be predicted. In this regard, to gain insights into the influence of BCAs on PAT accumulation, it was necessary to rely on a precise assessment of the pathogen biomass in infected apple tissues so that PAT accumulation could refer to the actual amount of fungal biomass responsible for its biosynthesis. For this purpose, we used and validated in our experimental conditions a qPCR method that was developed by Tannous et al. (2015). This method was reliable, accurate and specific for the quantification of the DNA contents and thus biomass of strains PY and FS7 of *P. expansum* in the presence or absence of the BCAs (**Figure 5**). In fact, the primers patF-F/patF-R designed against the *P. expansum* gene patF did not produce amplicons with genomic DNA samples from apple as already demonstrated by Tannous et al. (2015) and with genomic DNAs from the biocontrol yeasts (**Figure 3**). Furthermore, our results confirm the findings by Tannous et al. (2015) regarding the existence of a linear relationship between the pathogenic fungal DNA and PAT accumulation (**Supplementary Figure S1**).

For our assessment of the yeasts *R. mucilaginosa* 3617 and *R. kratochvilovae* LS11 for their biocontrol activities and influences on PAT accumulation in Fuji apples infected with strains PY and FS7, we used the highest concentration of yeast (10^8^ cells mL^-1^) that Zhu et al. (2015b) used in their study and that caused the highest level of PAT contamination in the same apple cultivar. Furthermore, we prolonged the total incubation time of the infected apples in our investigation to 9 days, which is 2 days longer than that used in the previous study on *R. paludigenum*. As expected, the two BCAs exerted antagonistic activities against blue mold decay caused by the two strains of *P. expansum*, both in terms of disease incidence and mean lesion diameter, (**Figure 1B**). The more robust protection exerted by the BCAs against *P. expansum* PY most likely reflects the lower aggressiveness of this strain as compared to FS7, which infected almost all the inoculated apples on day 5 (**Figure 1B**) and caused the formation of wider lesions than PY at all tested time points (**Figures 1C,D**). In this study, we evaluated as the aggressiveness of the *P. expansum* strains based on the disease incidence and symptoms rather than the biomass growth of the fungus in the infected fruit tissue. The higher disease incidence and symptoms caused by strain FS7 (**Figure 1B**) were accompanied by slightly but significantly lower *P. expansum* DNA content than strain PY at 7 and 9 days of apple storage (**Figure 5A** and **Supplementary Figures S1**–**S8**). On the other hand, the higher levels of disease and lower biomass growth of FS7 were also associated with a higher PAT accumulation at all the tested time points (**Figures 2A,B**). The assessment of the role of PAT in the interaction between apple and *P. expansum* is beyond the scope of the present study. However, this role has recently been a matter of debate (Sanzani et al., 2012; Barad et al., 2014; Ballester et al., 2015; Li et al., 2015). Our results seem to suggest that PAT could be involved in the aggressiveness of *P. expansum* (defined as the disease incidence and development of symptoms rather than the fungal biomass growth). However, a very interesting paper by Snini et al. (2016) showed that PAT is a cultivar-dependent aggressiveness factor, but it does not contribute to the aggressiveness of *P. expansum*, in terms of increase rate of lesion diameters and rotting volume, in the apple cultivar, Fuji, used in this study. Therefore, a standardized definition of the aggressiveness of *P. expansum* is needed.

Importantly, both LS11 and 3617 caused significant decreases of PAT accumulation for up to 9 days of storage with both *P. expansum* strain PY and *P. expansum* strain FS7, the higher PAT producer. These results concur with our previous results and those of other authors (Castoria et al., 2005; Lima et al., 2011; Yang et al., 2015). The decreases in PAT accumulation were generally more pronounced with the BCA LS11, especially at the higher levels of PAT contamination. This could be due to the higher resistance of LS11 to PAT as compared to strain 3617 (Castoria et al., 2005, 2011), to differences in yeast survival in the infected tissue, and/or to differences in the *in vivo* PAT-degrading capabilities of the two BCAs, although these degrading capabilities have insofar been demonstrated only *in vitro* (Castoria et al., 2005, 2011; Yang et al., 2015). The development of a qPCR method for the quantitation of the biomass of these biocontrol yeast strains and a method for the assessment of their *in vivo* PAT degradation capabilities are in progress.

Interestingly, the presence of the BCAs in diseased apples led to increases in the specific mycotoxigenic activity of strains PY and FS7 (**Figure 6**). This could result from the stress caused to the fungal pathogens by the antagonistic activities of the BCAs. In addition, some BCAs have been reported to resist to oxidative stress caused by reactive oxygen species (ROS) (Castoria et al., 2003), but also produce and induce ROS generation in apple wounds (Macarisin et al., 2010). These ROS have been suggested to trigger the onset of secondary metabolism and mycotoxin biosynthesis in fungi (Reverberi et al., 2010; Montibus et al., 2015).

To our knowledge, this is the first report that evaluated the effect of BCAs (i) on the growth rate in stored fruits of mycotoxigenic fungal pathogens quantitatively measured through qPCR, and (ii) on the specific rate of mycotoxin synthesis.

## Conclusion

The biocontrol yeasts *R. mucilaginosa* 3617 and *R. kratochvilovae* LS11 exerted antagonistic activities against blue mold and limited PAT accumulation in apples infected by two strains of *P. expansum* from different geographic locations. Nevertheless, the specific mycotoxigenic activity of the *P. expansum* strains was increased, and at a different level, by the presence of the BCAs. This suggests that different BCAs might have different effects on PAT accumulation that could also depend on the *P. expansum* strain and the host cultivar, possibly leading to undesired side effects such as the increase of PAT accumulation recorded by Zhu et al. (2015a). PAT accumulation was also reported to be stimulated by fungicides (Paterson, 2007), but unlike fungicides, BCAs are “active elements” that are potentially able to interfere with mycotoxin synthesis and/or degrade the mycotoxins *in vivo*. Therefore, further studies are needed for the evaluation of other BCA/apple cultivar/*P. expansum* strain/storage condition combinations.

## Author Contributions

HZ and RC designed the experiments and revised the manuscript; XfZ and QY performed the experiments and analyzed results; XyZ provided direction in experimental methods and revised the manuscript; MA revised the manuscript; GI provided suggestions for the PCR analyses, for the experimental design and revised the manuscript.

## Conflict of Interest Statement

The authors declare that the research was conducted in the absence of any commercial or financial relationships that could be construed as a potential conflict of interest.

## Supplementary Material

FIGURE S1 Correlation between *Penicillium expansum* biomass (expressed as ng of DNA/μg of decayed tissue) and patulin content (μg/g of decayed tissue) in artificially infected apples treated with the following microorganisms: PePY **(A)**; RkLS11+PePY **(B)**; Rm3617+PePY **(C)**; PeFS7 **(D)**; RkLS11+PeFS7 **(E)**; Rm3617+PeFS7 **(F)**. PePY, *Penicillium expansum* strain PY; PeFS7, *P. expansum* strain FS7; RkLS11, *Rhodotorula kratochvilovae* strain LS11; Rm3617, *R. mucilaginosa* strain 3617.

FIGURE S2 Correlation between lesion diameter (mm) and *P. expansum* biomass (expressed as ng of DNA/μg of decayed tissue) in artificially infected apples treated with the following microorganisms: PePY **(A)**; RkLS11+PePY **(B)**; Rm3617+PePY **(C)**; PeFS7 **(D)**; RkLS11+PeFS7 **(E)**; Rm3617+PeFS7 **(F)**. PePY, *Penicillium expansum* strain PY; PeFS7, *P. expansum* strain FS7; RkLS11, *Rhodotorula kratochvilovae* strain LS11; Rm3617, *R. mucilaginosa* strain 3617.

FIGURE S3 Correlation between patulin content (μg/g of decayed apple tissue) and lesion diameter (mm) in artificially infected apples inoculated treated with the following microorganisms: PePY **(A)**; RkLS11+PePY **(B)**; Rm3617+PePY **(C)**; PeFS7 **(D)**; RkLS11+PeFS7 **(E)**; Rm3617+PeFS7 **(F)**. PePY, *Penicillium expansum* strain PY; PeFS7, *P. expansum* strain FS7; RkLS11, *Rhodotorula kratochvilovae* strain LS11; Rm3617, *R. mucilaginosa* strain 3617.

FIGURE S4 Time course of disease incidence (% of infected wounds) in artificially inoculated apples stored at 20°C. Bars represent the mean values from two experiments ± standard deviations. Bars with ^∗^ indicate significant difference (*P* < 0.05). PePY, *Penicillium expansum* strain PY; PeFS7, *P. expansum* strain FS7.

FIGURE S5 Time course of lesion diameter (mm) in artificially inoculated apples stored at 20°C. Bars represent the mean values from two experiments ± standard deviations. Bars with ^∗^ indicate significant difference (*P* < 0.05). PePY, *Penicillium expansum* strain PY; PeFS7, *P. expansum* strain FS7.

FIGURE S6 Time course of patulin contamination (μg/g of decayed apple tissue) in apples artificially infected by PePY and PeFS7 during storage at 20°C. Bars represent the mean values from two experiments ± standard deviations. Bars with ^∗^ indicate significant difference (*P* < 0.05). PePY, *Penicillium expansum* strain PY; PeFS7, *P. expansum* strain FS7.

FIGURE S7 Time course of *P. expansum* biomass development (ng DNA/μg of decayed apple tissue) in apples artificially infected by PePY and PeFS7 during storage at 20°C. Bars represent the mean values from two experiments ± standard deviations. Bars with ^∗^ indicate signficant difference (*P* < 0.05). PePY, *Penicillium expansum* strain PY; PeFS7, *P. expansum* strain FS7.

FIGURE S8 Time course of specific mycotoxigenic activity (ng patulin/μg of fungal DNA) of strains PY and FS7 of *Penicillium expansum* in infected apples stored at 20°C. Bars represent the mean values from two experiments ± standard deviations. Bars with ^∗^ indicate significant difference (*P* < 0.05). PePY, *Penicillium expansum* strain PY; PeFS7, *P. expansum* strain FS7.
