# Supplementary figures and images for: Biocontrol Agents Increase the Specific Rate of Patulin Production by Penicillium expansum but Decrease the Disease and Total Patulin Contamination of Apples
Source: Front Microbiol. 2017 Jun 30;8:1240. doi: 10.3389/fmicb.2017.01240 (PMC5492354; doi:10.3389/fmicb.2017.01240)

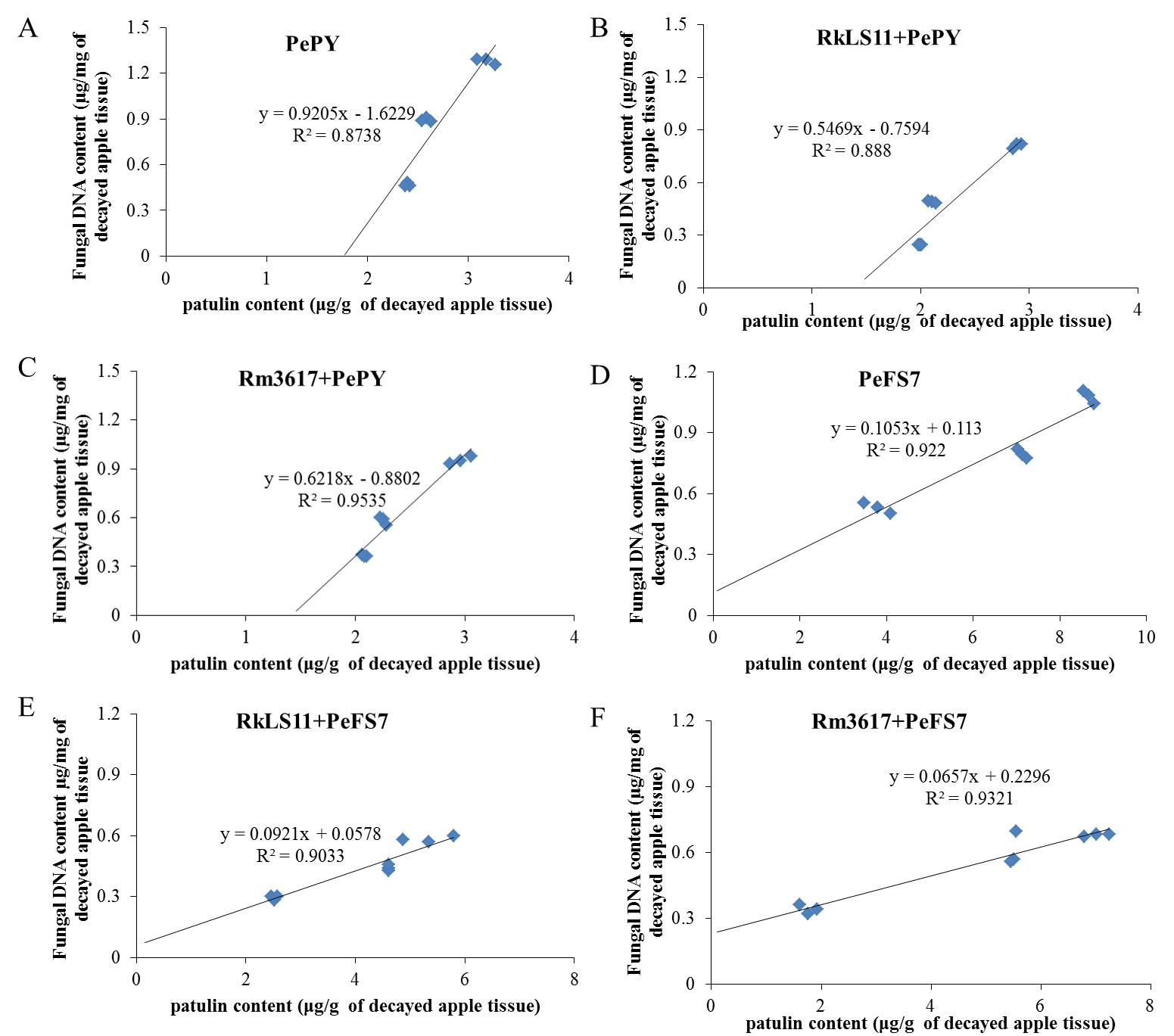

Supplement: FIGURE S1 — Correlation between Penicillium expansum biomass (expressed as ng of DNA/μg of decayed tissue) and patulin content (μg/g of decayed tissue) in artificially infected apples treated with the following microorganisms: PePY (A); RkLS11+PePY (B); Rm3617+PePY (C); PeFS7 (D); RkLS11+PeFS7 (E); Rm3617+PeFS7 (F). PePY, Penicillium expansum strain PY; PeFS7, P. expansum strain FS7; RkLS11, Rhodotorula kratochvilovae strain LS11; Rm3617, R. mucilaginosa strain 3617. [file Image_1.JPEG]

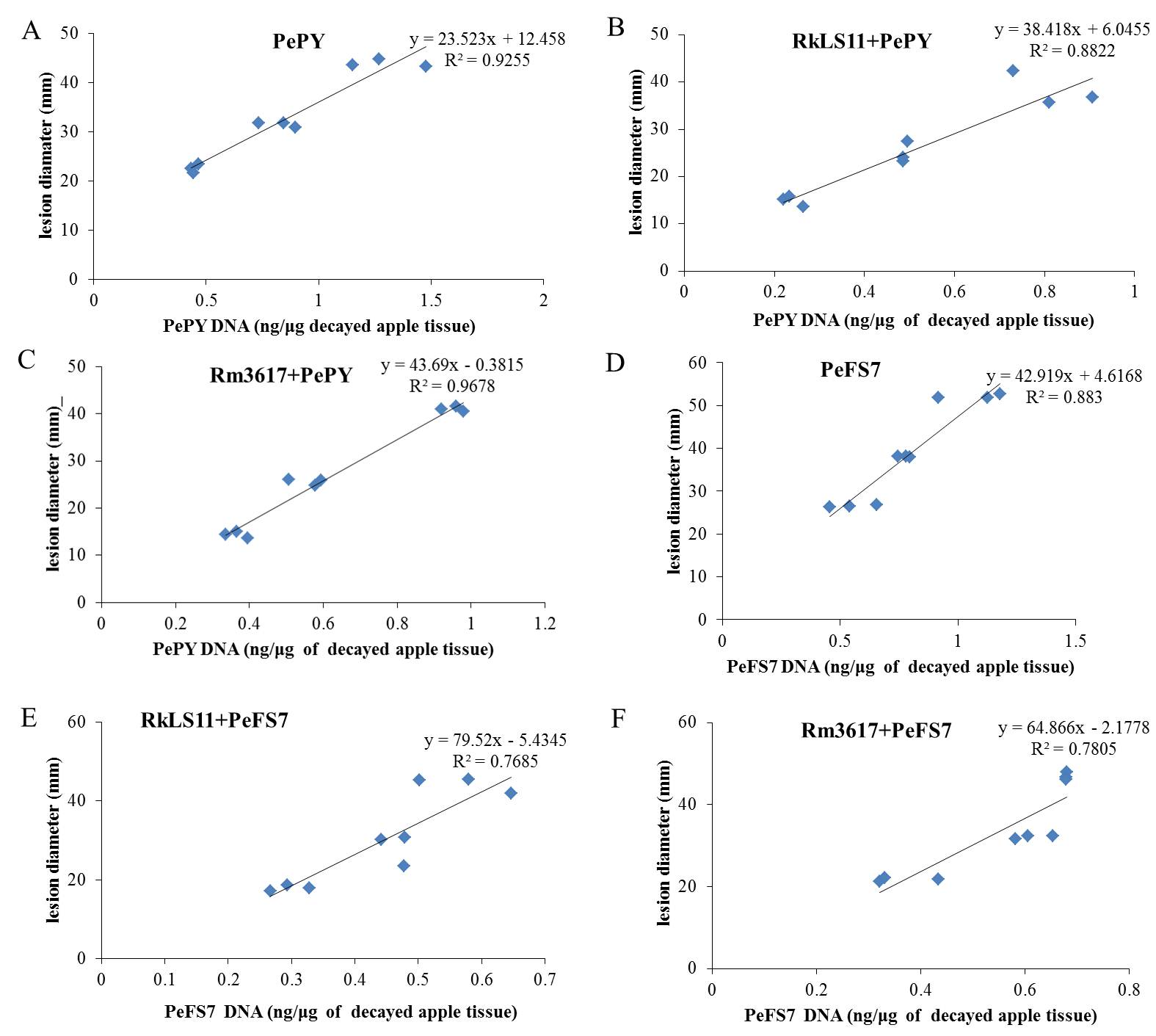

Supplement: FIGURE S2 — Correlation between lesion diameter (mm) and P. expansum biomass (expressed as ng of DNA/μg of decayed tissue) in artificially infected apples treated with the following microorganisms: PePY (A); RkLS11+PePY (B); Rm3617+PePY (C); PeFS7 (D); RkLS11+PeFS7 (E); Rm3617+PeFS7 (F). PePY, Penicillium expansum strain PY; PeFS7, P. expansum strain FS7; RkLS11, Rhodotorula kratochvilovae strain LS11; Rm3617, R. mucilaginosa strain 3617. [file Image_2.JPEG]

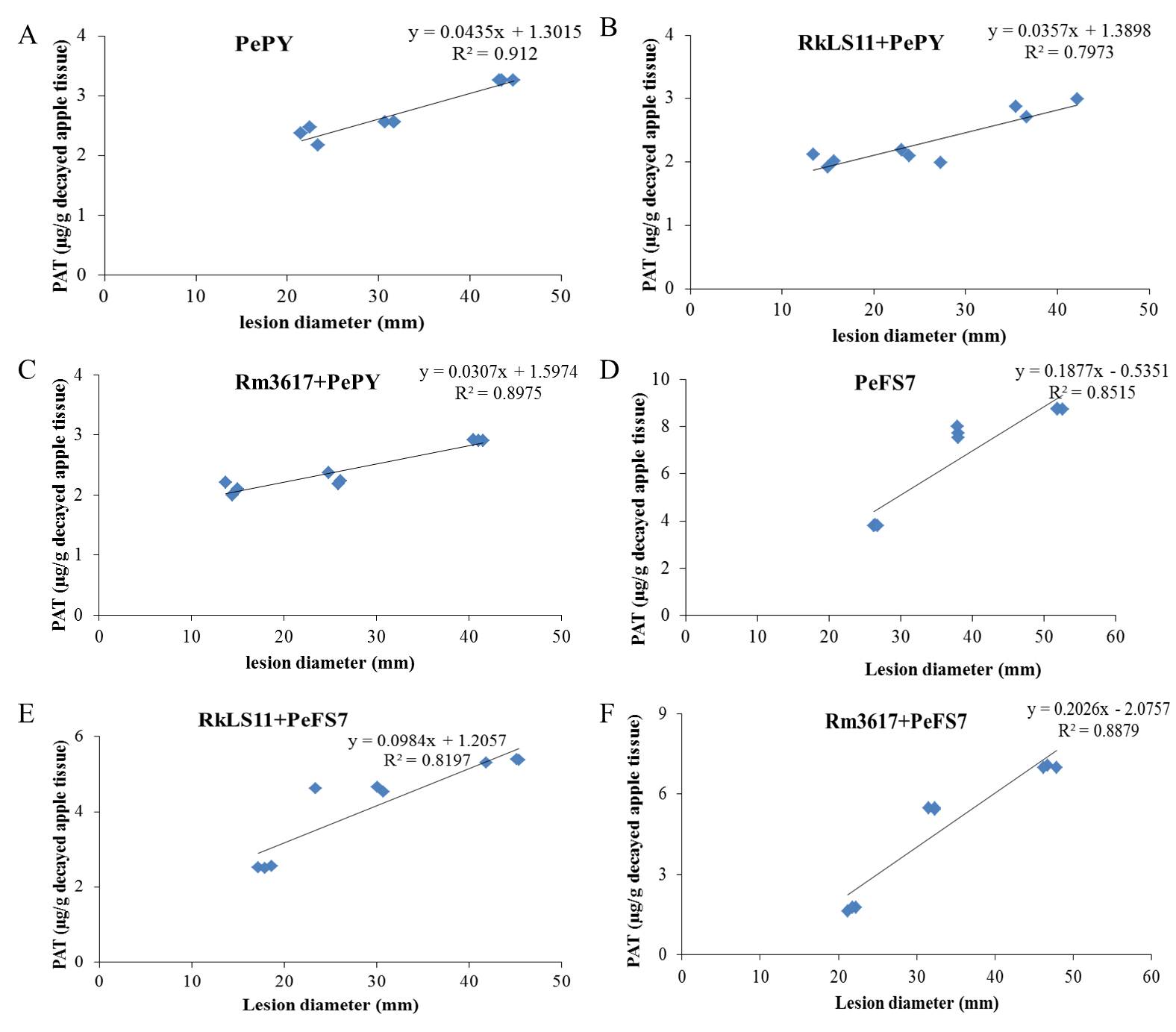

Supplement: FIGURE S3 — Correlation between patulin content (μg/g of decayed apple tissue) and lesion diameter (mm) in artificially infected apples inoculated treated with the following microorganisms: PePY (A); RkLS11+PePY (B); Rm3617+PePY (C); PeFS7 (D); RkLS11+PeFS7 (E); Rm3617+PeFS7 (F). PePY, Penicillium expansum strain PY; PeFS7, P. expansum strain FS7; RkLS11, Rhodotorula kratochvilovae strain LS11; Rm3617, R. mucilaginosa strain 3617. [file Image_3.JPEG]

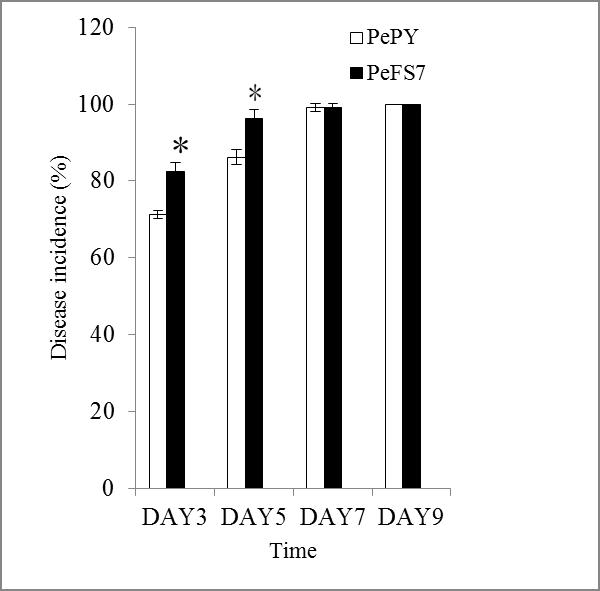

Supplement: FIGURE S4 — Time course of disease incidence (% of infected wounds) in artificially inoculated apples stored at 20°C. Bars represent the mean values from two experiments ± standard deviations. Bars with ∗ indicate significant difference (P < 0.05). PePY, Penicillium expansum strain PY; PeFS7, P. expansum strain FS7. [file Image_4.JPEG]

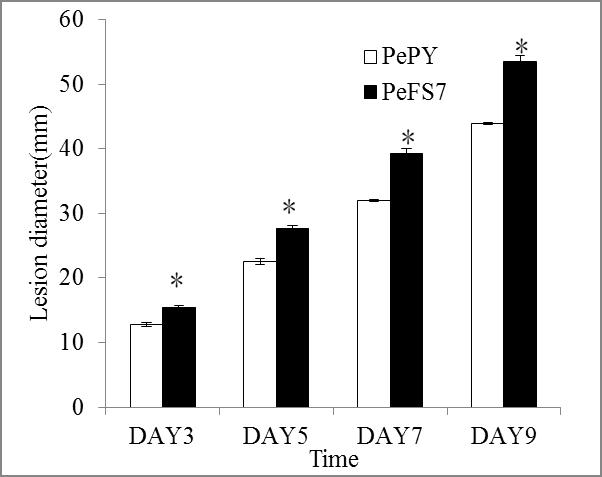

Supplement: FIGURE S5 — Time course of lesion diameter (mm) in artificially inoculated apples stored at 20°C. Bars represent the mean values from two experiments ± standard deviations. Bars with ∗ indicate significant difference (P < 0.05). PePY, Penicillium expansum strain PY; PeFS7, P. expansum strain FS7. [file Image_5.JPEG]

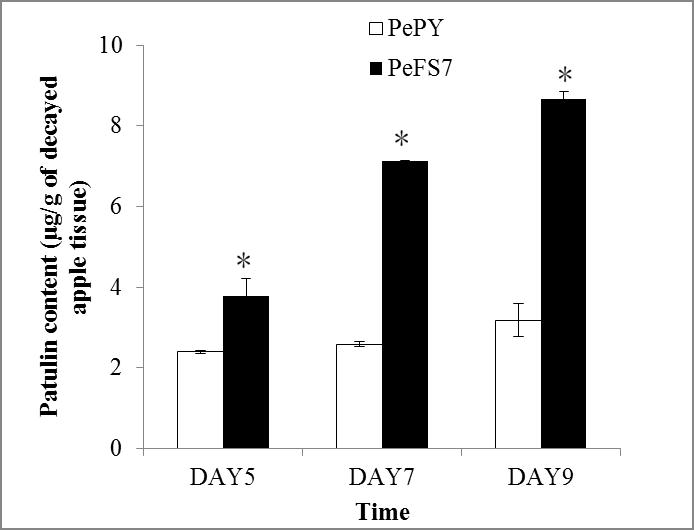

Supplement: FIGURE S6 — Time course of patulin contamination (μg/g of decayed apple tissue) in apples artificially infected by PePY and PeFS7 during storage at 20°C. Bars represent the mean values from two experiments ± standard deviations. Bars with ∗ indicate significant difference (P < 0.05). PePY, Penicillium expansum strain PY; PeFS7, P. expansum strain FS7. [file Image_6.JPEG]

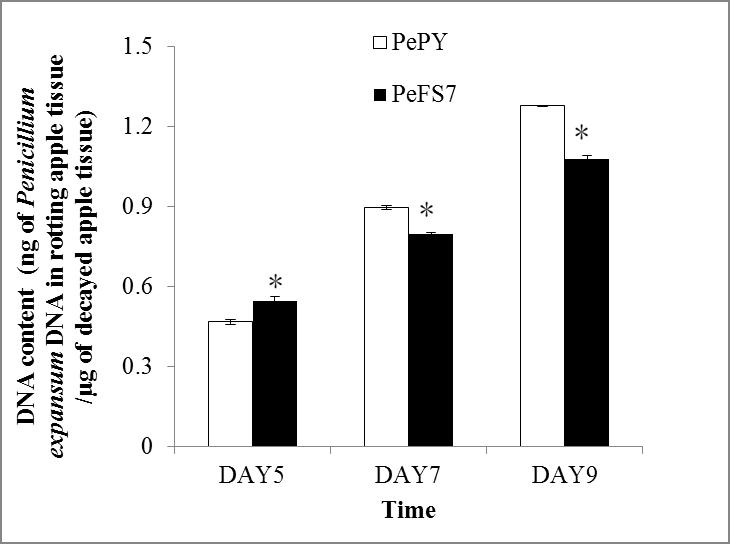

Supplement: FIGURE S7 — Time course of P. expansum biomass development (ng DNA/μg of decayed apple tissue) in apples artificially infected by PePY and PeFS7 during storage at 20°C. Bars represent the mean values from two experiments ± standard deviations. Bars with ∗ indicate signficant difference (P < 0.05). PePY, Penicillium expansum strain PY; PeFS7, P. expansum strain FS7. [file Image_7.JPEG]

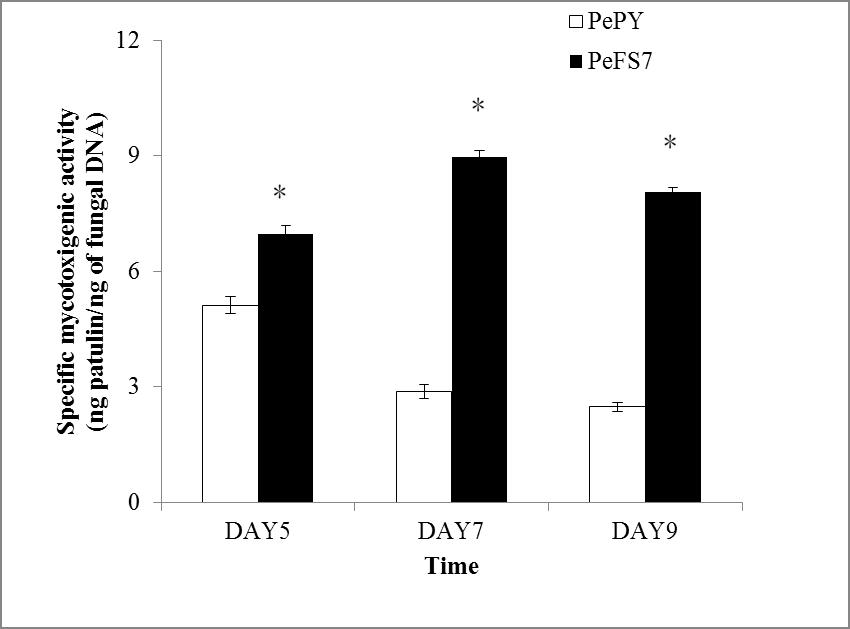

Supplement: FIGURE S8 — Time course of specific mycotoxigenic activity (ng patulin/μg of fungal DNA) of strains PY and FS7 of Penicillium expansum in infected apples stored at 20°C. Bars represent the mean values from two experiments ± standard deviations. Bars with ∗ indicate significant difference (P < 0.05). PePY, Penicillium expansum strain PY; PeFS7, P. expansum strain FS7. [file Image_8.JPEG]
